# Characterization of a Novel Oxidative Stress Responsive Transcription Regulator in *Mycobacterium bovis*

**DOI:** 10.3390/biomedicines12081872

**Published:** 2024-08-16

**Authors:** Qiang Jiang, Rong Hu, Feng Liu, Feng Huang, Lei Zhang, Hua Zhang

**Affiliations:** 1 College of Life Science and Technology, Huazhong Agricultural University, Wuhan 430070, China; jq12025@163.com (Q.J.);; 2 College of Veterinary Medicine, Huazhong Agricultural University, Wuhan 430070, China

**Keywords:** *Mycobacterium bovis*, transcription regulator, oxidative stress, BCG_3893c

## Abstract

The antioxidant defense is critical for the survival of intracellular pathogens such as *Mycobacterium tuberculosis* complex (MTBC) species, including *Mycobacterium bovis,* which are often exposed to an oxidative environment caused by reactive oxygen species (ROS) in hosts. However, the signaling pathway in mycobacteria for sensing and responding to oxidative stress remains largely unclear. In this study, we characterize a TetR-type transcription regulator BCG_3893c, designated AotM, as a novel redox sensor in *Mycobacterium bovis* that increases mycobacterial tolerance to oxidative stress. AotM is required for the growth of *M. bovis* in the presence of 1 mM hydrogen peroxide. Loss of the *aotM* gene leads to altered transcriptional profiles with 352 genes significantly up-regulated and 25 genes significantly down-regulated. AotM recognizes a 14-bp palindrome sequence motif and negatively regulates the expression of a FAD-dependent oxidoreductase encoded by *bcg_3892c*. Overexpression of BCG_3892c increases intracellular ROS production and reduces the growth of *M. bovis*. In summary, we propose that AotM enhances the mycobacterial resistance against oxidative stress probably by inhibiting intracellular ROS production. Our findings reveal a novel underlying regulatory mechanism behind mycobacterial oxidative stress adaptation.

## 1. Introduction

Tuberculosis (TB) is a serious zoonotic disease caused by the *Mycobacterium tuberculosis* complex (MTBC) that kills millions of people worldwide annually [1,2]. *M. tuberculosis* and *M. bovis* are the two major members of the MTBC. They share almost identical genomic sequences (identity > 99.95%) [1,3,4] and, therefore, cause very similar symptoms in humans and animals [5,6]. These pathogens are mainly transmitted by aerosols, which can reach the lungs for bacterial colonization. They could infect a variety of cells, including neutrophils, macrophages, and dendritic cells [7,8], and cause granulomas in the hosts [9]. During the course of infection, these intracellular pathogens are exposed to a variety of host-mediated stresses, in which reactive oxygen species (ROS) is one of the primary antimicrobial agents produced by phagocyte oxidase in macrophages [10]. Excess ROS could induce damage to the essential cellular components such as proteins, lipids, and nucleic acids [11]. Therefore, the antioxidant defense becomes particularly important for the survival of intracellular pathogens such as *M. tuberculosis*, which has evolved multiple strategies to resist ROS, extracellular and intracellular. For example, the mycobacterial cell wall serves as the first defense barrier against ROS, and the low permeability of the cell wall can block the entry of environmental toxic substances into the bacterial cell [12]. Also, Mtb expresses many antioxidant proteins including superoxide dismutases SodA/SodC [13], catalase KatG [14], peroxidase AhpC [15], thioredoxins (Trx) [16], etc., to eliminate intracellular ROS. In addition, the histone-like Lsr2 with ferroxidase activity was found to be involved in the detoxification of iron and hydrogen peroxide [17]. Although many proteins involved in antioxidant process have been reported in mycobacteria, the molecular mechanism and the signaling pathways by which mycobacteria adaptively promote antioxidant regulation remains largely unclear.

The transcription regulators that can directly sense ROS and regulate bacterial antioxidant growth have been reported in many bacteria. For example, OxyR is a well-studied redox sensor in *Escherichia coli*. Sensing oxidative signals by the C-terminal cysteine residues, OxyR could form intramolecular disulfide bonds [18,19] and promote expression of the antioxidant genes, including *katG*, *ahpCF*, *gorA*, etc., to protect the cells from the toxicity of hydrogen peroxide [18,20,21]. Similarly, OhrR regulates the expression of organic hydroperoxide reductase (*ohrA*) in *Bacillus subtilis* for bacterial survival in toxic environments by the N-terminal cysteine-mediated oxidative signal sensing [22,23,24]. In mycobacterial species, SoxR senses the presence of superoxide via its iron-sulfur cluster (2Fe-2S) [25,26]. SoxR would undergo monovalent oxidation to form a reactive oxidation state, which activates the expression of *soxS*. SoxS protein would further activate the superoxide dismutase, etc., for detoxification of the superoxide [25]. Recently, CmtR, a Cadmium/Plumbum-sensing transcription regulator, was reported to respond to oxidative signals and regulate the antioxidant growth of *M. bovis*. It can disarm the zinc uptake regulator Zur, which represses the expression of the *esx-3* operon, promoting accumulation of zinc and detoxication of ROS in mycobacterial cells [27].

In this study, we characterized AotM/BCG_3893c, a TetR-type transcription regulator, as a novel redox sensor in *Mycobacterium bovis* that is required for mycobacterial growth in the presence of hydrogen peroxide. AotM responds to the oxidative signal and promotes the expression of the antioxidant genes. Meanwhile, AotM might restrict intracellular ROS production by repressing the expression of *bcg_3892c*, which encodes a dehydrogenase. Our findings indicate the existence of a novel antioxidant regulation pathway and provide new insights into mycobacterial adaptation to oxidative stress.

## 2. Materials and Methods

### 2.1. Bacterial Strains, Primers and Media

The strains and plasmids used in the experiments are listed in Tables S1 and S2. The primers used in this study are shown in Table S3. LB liquid medium (Sinopharm Chemical Reagent Co., Ltd., Shanghai, China), LB solid medium (Sinopharm Chemical Reagent Co., Ltd., Shanghai, China), 7H9 medium (Becton, Dickinson and Company, Franklin Lakes, NJ, USA), and 7H10 medium (Becton, Dickinson and Company, Franklin Lakes, NJ, USA) were used in this study.

### 2.2. Protein Expression and Purification

The *aotM* gene was amplified from the genomic DNA of *Mycobacterium bovis* BCG by PCR using specific primers (Table S3). Then, the *aotM* gene was cloned into pET28a vector for protein expression in *E. coil* BL21 (DE3) strain. One liter of Luria-Bertani (LB) medium containing 30 μg/mL Kanamycin was used for producing the His6-tagged AotM protein. Cells were grown at 37 °C to OD_600_ were approximately 0.8 to 1.0. Then, 0.5 mM isopropyl thiogalactoside (IPTG) was added into the culture media to induce expression of AotM protein for 4 h. Afterward, cells were harvested by centrifugation and lysed by sonication in ice-cold lysis buffer (150 mM NaCl, 30 mM Tris, 1 mM PMSF [pH 7.5]). Cell lysate was clarified by centrifugation, and the proteins were purified on Ni^2+^ affinity columns as described previously [28]. The elution was then dialyzed overnight and stored at −80 °C. The protein concentration was determined by Coomassie Brilliant Blue assay.

### 2.3. Real-Time Fluorescence Quantitative PCR Assay

All the *M. bovis* BCG strains were cultured in 7H9 medium to OD_600_ of 0.7, RNA was extracted by RNA extraction kit (Beijing Aidlab Biotechnologies Co., Ltd., Beijing, China), 0.8% agarose gel was used to detect the purity and integrity of RNA samples. Real-time fluorescence quantitative PCRs (RT-qPCRs) were performed using a 20 μL reaction mixture with the following ingredients: 10 μL 2× SYBR qPCR Mix kit (Aidlab, Beijing, China), 200 nM concentration of genes primer pairs (Table S3), and 2 μL volume of the immunoprecipitated and purified DNA samples. Each reaction was performed in triplicate. The reactions were reacted in a CFX96 instrument (Bio-Rad, Hercules, CA, USA) using the following protocol: 95 °C for 5 min; 35 cycles of 95 °C for 30 s, 60 °C for 30 s, and 72 °C for 30 s. The Bio-Rad CFX Manager v.2.1 software was used to analyze the data. The expression levels of different genes were normalized by *sigA*, and RT-qPCR data were calculated using the 2^−ΔΔCt^ method [29]. For statistical analysis, two-tailed Student’s *t*-tests were performed.

### 2.4. Growth Curves Assays

The growth curves of the *M. bovis* BCG strains were determined as previously described [30]. For the initial culturing, all the strains, including the wild-type BCG (WT BCG), the *aotM* deletion mutant (Δ*aotM*), the *aotM* overexpressing strain (WT/pMV261-*aotM*), and the *aotM* complementary strain (Δ*aotM + aotM)* were grown in 7H9 media till OD_600_ reached 1.0. Then, all the strains were inoculated into 10 mL of fresh 7H9 medium at an initial OD_600_ of 0.01 with and without 1 mM H_2_O_2_. The OD_600_ of the cultures was determined every 24 h. For statistical data, growth curves were plotted in Graphpad Prism 8 (8.0.1), three biological replicates were set for each group, and error bars were calculated.

### 2.5. DNA Substrate Preparation and Electrophoretic Mobility Shift Assay (EMSA)

The DNA fragments, including *aotMp*, *ppsAp*, *BCG_3892cp,* and *BCG_3894p* for electrophoretic mobility shift assay (EMSA), were amplified by PCR from the genome of *M. bovis* BCG using the specific primers (Table S3). The oligonucleotides (P1 to P4) were directly synthesized and were mixed with the equal molar concentration of their reverse complementary oligonucleotides (Table S3). Then, the oligonucleotide mixtures were heated at 95 °C for 5 min until annealed by natural cooling. The DNA-binding assays were performed according to a previous procedure [28]. Briefly, the reactions (20 μL) for measuring mobility shift contained labeled DNA substrates and increasing concentrations of the protein (as indicated in the legend of the corresponding figure) and were incubated in EMSA buffer (50 mM Tris–HCl, pH 7.5, 10 mM MgCl_2_, 1 mM DTT and 50 mM NaCl) at 4 °C for 30 min and then subjected to 5% native PAGE. Electrophoresis was performed at 150 V at room temperature for 1.5 h in 0.5× Trisborate-EDTA buffer. Images of gels were acquired using a Typhoon Scanner (GE Healthcare, Chicago, IL, USA).

### 2.6. ChIP Assay

The chromatin immunoprecipitation (ChIP) experiment was performed as previously described [31]. The *M. bovis* BCG cells were grown in 100 mL of 7H9 medium up to an OD_600_ of 1.0. Then, the cells were fixed with 1% formaldehyde for 20 min, and the reaction was ended with 0.125 M glycine for 5 min. The cross-linked cells were harvested and resuspended in 1 mL of TBSTT (TBS, 0.2% Triton X-100, 0.05% Tween-20). The sample was sonicated on ice, and the average size of the DNA fragments was approximately 0.5 kb. A 100 μL sample of the extract was saved as the input fraction. The remaining 900 μL was then incubated with 10 μL of the AotM antibody or pre-immune serum at 4 °C for 3 h. The complexes were immunoprecipitated with 20 μL 50% Protein A agarose (Becton, Dickinson and Company, Franklin Lakes, NJ, USA) under rotation at 4 °C for 1 h. The immunocomplexs were recovered by centrifugation and resuspended in 100 μL TE buffer (20 mM Tris–HCl pH 7.8, 10 mM EDTA, 0.5% SDS). The cross-linking was reversed at 65 °C for 6 h. The sample DNA was purified and amplified by PCR with Platinum Taq (Invitrogen, Carlsbad, CA, USA). Each experiment was performed in duplicate and was repeated twice. The amplification protocol includes one denaturation step of 5 min at 95 °C, then 32 cycles of 1 min at 95 °C, 1 min at 60 °C, and 1 min at 72 °C.

### 2.7. DNA Footprinting Assay

The *aotMp* DNA fragment was amplified with the FAM-labeled primers. The purified DNA fragment was added to the reaction and mixed with the increased amount of AotM at room temperature for 30 min for the EMSA experiment. All of the mixtures were treated with 1 unit DNase I (Fermentas, Beijing, China) at 37 °C for 2 min and 30 s as previously described [32,33]. The results were analyzed with Applied Biosystems 37030XL DNA analyzer (Tsingke, Wuhan, China).

### 2.8. β-Galactosidase Activity Assay

The galactosidase activity assays were performed in accordance with previously described procedures [30]. The target and control promoters were cloned into pMV261 backbone, and then the reporter gene *lacZ* was cloned behind the promoters. The plasmids were transformed into the *Escherichia coli* DH5α strains to obtain their corresponding recombinant reporter strains. The strains containing the *lacZ* reporter vectors (Table S2) were used in the experiments [34]. All strains were cultured in LB medium at 37 °C to the mid-log phase. Then, the bacterial cells were harvested and washed twice with PBS and afterward were streaked on the LB plates supplemented with 30 μg/mL kanamycin, 0.1% IPTG, and 40 μg/mL X-gal for culturing at 37 °C overnight. The β-galactosidase measurements were performed as described previously [35].

### 2.9. Determination of Intracellular ROS Content

The intracellular ROS content was determined by the dichloro-dihydro-fluorescein diacetate (DCFH-DA) assay [36] with modifications. The wild-type BCG strain and the BCG_3892c overexpressing strain were cultured in 50 mL of 7H9 medium to an OD_600_ of 1.2, and then 10 mL of the bacterial culture was centrifuged at 10,000× *g* for 1 min, and the supernatant was discarded. The cells were washed with 5 mL of PBS three times and then resuspended in 500 μL PBS. Then the cells were incubated with 100 μM DCFH-DA at 37 °C in the dark for 30 min. The cells were washed two times with PBS to remove the extracellular DCFH-DA. Subsequently, the fluorescence signal in the bacteria was detected by flow cytometry. Finally, the level of fluorescence signal detected in the two strains was statistically analyzed and plotted using GraphPad Prism 8 (8.0.1).

### 2.10. Transcriptomic Analysis

The transcriptomic analysis was conducted as previously described [30]. The strains were grown in 100 mL 7H9 media at 37 °C with 160 rpm shaking. The cells were cultured to the mid-logarithmic phase (optical density at 600 nm [OD_600_], 1.0) and were harvested. Each strain was cultured in three biological replicates. The total RNA was isolated using RNeasy mini kit (Qiagen, Hilden, Germany). Strand-specific libraries were prepared using the TruSeq stranded total RNA sample preparation kit (Illumina, San Diego, CA, USA) following the manufacturer’s instructions. Library construction and sequencing were performed at Novogene Biotechnology Corporation (Tianjin, China).

## 3. Results

### 3.1. aotM Gene Is Required for Mycobacterium bovis to Resist Oxidative Stress

**Figure 1:**
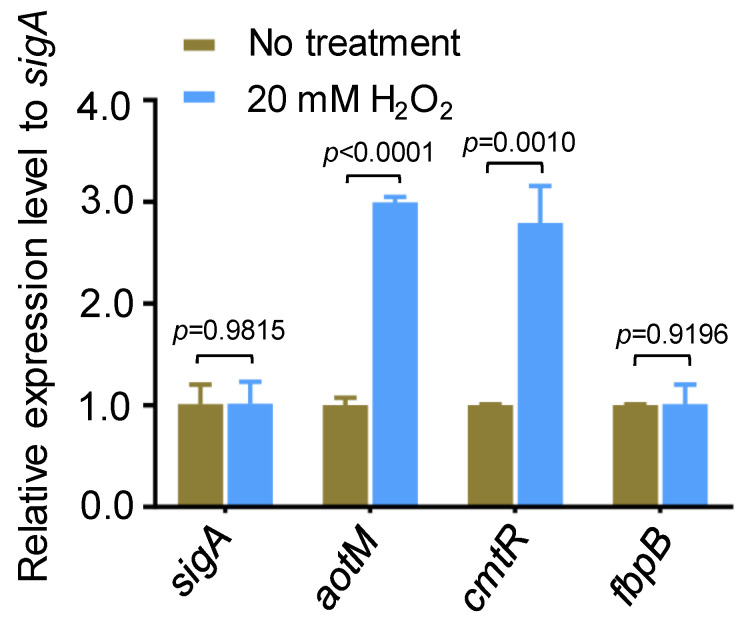
The expression of *aotM* gene is induced by H_2_O_2_ stress. RT-qPCR was performed to detect the expression of *aotM* in wild-type *M. bovis* BCG strains treated with 20 mM H_2_O_2_. The relative gene expression levels were normalized using the *sigA* gene as the internal reference. *cmtR* and *fbpB* were used as the positive control and the negative control, respectively. Error bars represent the standard deviation of the three biological replicates. A two-tailed unpaired Student’s *t*-test was used for statistical analysis using GraphPad Prism 8. *p*-values are indicated.

In an oxidative stress experiment in *Mycobacterium bovis*, we noticed that *aotM* (*BCG_3893c*), encoding a TetR family transcription regulator, could be induced by hydrogen peroxide. With the treatment of 20 mM H_2_O_2_ for 1.5 h, the transcription level of *aotM* was increased by 3-fold compared with the untreated strain (Figure 1). *cmtR*, a H_2_O_2_-induced transcription regulator [27] as the positive control, was up-regulated approximately 2.7-fold, and the oxidative stress unrelated gene *fbpB* was unchanged (Figure 1). Our observation suggests that *aotM* could respond to the oxidative signal in *Mycobacterium bovis*.

**Figure 2:**
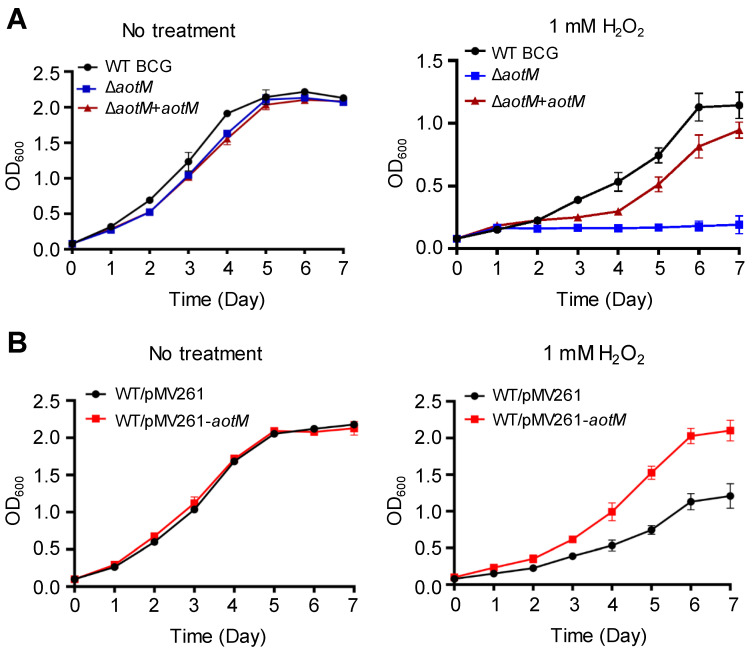
Growth assays of the *M. bovis* strains under H_2_O_2_ stress. (**A**) The wild-type *M. bovis* BCG strain (WT BGC), the *aotM* deletion mutant (Δ*aotM*) and the *aotM* complementary strain (*ΔaotM* + *aotM*) were grown in 7H9 media with (**right**) and without (**left**) 1 mM H_2_O_2_. (**B**) The WT strain (WT/pMV261) and the *aotM* overexpressing strain (WT/pMV261-*aotM*) were grown in 7H9 media with (**right**) and without (**left**) 1 mM H_2_O_2_. The initial OD_600_ of all cultures was 0.01. Error bars represent standard deviations from the mean values of three biological replicates.

To investigate the role of AotM, we constructed the Δ*aotM* deletion mutant using *Mycobacterium bovis* BCG. Deletion of *aotM* gene did not affect the growth of *M. bovis* BCG in the Middlebrook 7H9 liquid media (Figure 2A). In the presence of 1 mM H_2_O_2_, the growth of the Δ*aotM* deletion mutant was completely inhibited, while the wild-type *M. bovis* BCG strain, as well as the *aotM* complemented strain, were able to grow (Figure 2A), indicating that *aotM* gene is required for *M. bovis* BCG to resist H_2_O_2_ stress. Furthermore, overexpression of *aotM* gene in the WT BCG via *hsp60* promoter from the mycobacterial expression vector pMV261 (Figure S1) showed significantly increased growth in contrast to the WT BCG strain with the empty vector as the control in the presence of 1 mM H_2_O_2_ (Figure 2B), suggesting that *aotM* gene enhances H_2_O_2_ resistance of *M. bovis* BCG. Taken together, these results suggest that the transcription regulator AotM is required for the resistance of *M. bovis* to oxidative stress.

### 3.2. AotM Specifically Binds to Its Own Promoter Region

**Figure 3:**
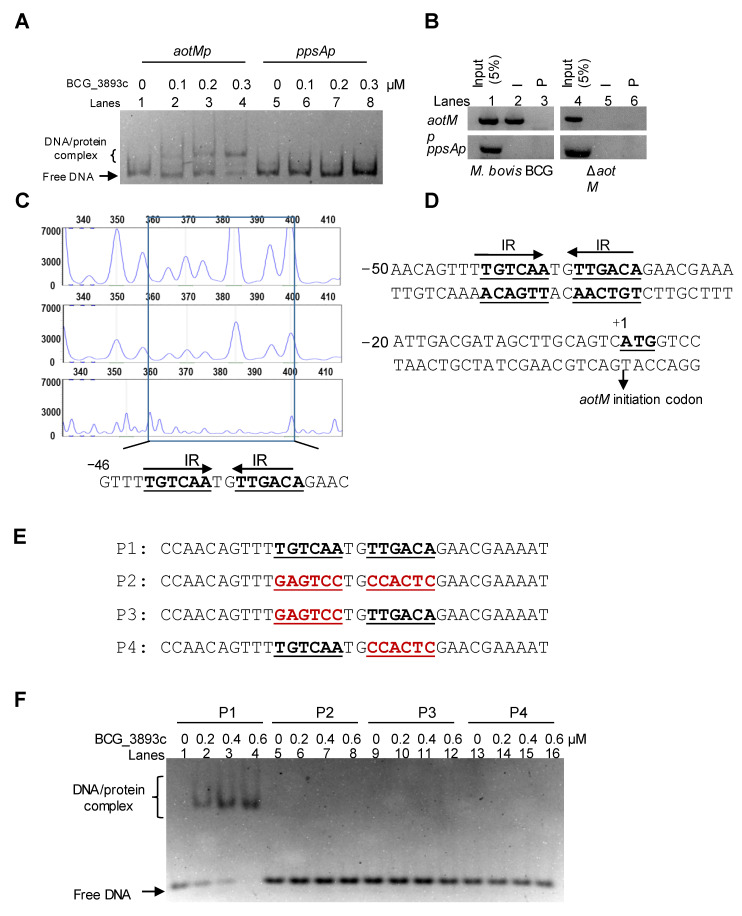
Analyses of the AotM binding motif. (**A**) EMSA assay for the DNA binding activity of AotM. The mixtures were electrophoresed in a 5% native polyacrylamide gel. The 100 ng *aotM* promoter (*aotMp*, 500 bp, lanes 1–4) and 100 ng negative control gene promoter (*ppsAp*, 500 bp, lanes 5–8) were incubated with different concentrations (0–0.3 µM) of AotM protein. (**B**) ChIP assays. The pre-immune (P) serum and the post-immune (I) serum were used in the ChIP. An mycobacterial promoter *ppsAp* was used as a negative control. (**C**) The DNase I footprinting assay. The protection of the *aotM* promoter DNA against DNaseI digestion by increasing amounts of AotM was evaluated. The sequences of the protected regions on the non-coding strand are marked in the box in the figures. (**D**) Sequence and structural characteristics of the promoter region protected by AotM. The regions protected by AotM are indicated with underlines; the translation start codon of AotM is indicated in bold. (**E**) The DNA binding motif with the designed IR mutated regions. The mutated regions are indicated in red. (**F**) EMSA of the short DNA substrates (34 bp), with or without the IR sequence. The mixtures were electrophoresed in a 5% native polyacrylamide gel. Each DNA substrate was co-incubated with 0–0.6 µM AotM protein.

To test the DNA binding activity of AotM, we performed an electrophoretic mobility shift assay (EMSA). The purified hexahistidine-tagged AotM protein was incubated with *aotM* upstream DNA (*aotMp*) or *ppsA* upstream DNA (*ppsAp*). As shown in Figure 3A, when 100 ng *aotMp* DNA fragment was co-incubated with increasing concentrations of AotM protein (0, 0.1, 0.2, and 0.3 μM), a specific and clear shift in the bands was observed (lanes 1–4). In contrast, AotM protein failed to cause shifts in the *ppsAp* DNA as the negative control (lanes 5–8), indicating that AotM specifically binds to its own promoter DNA region in vitro. Furthermore, in the chromatin immunoprecipitation (ChIP) assay, the complex of AotM protein and the *aotMp* DNA could be captured by the AotM antibody in the wild-type BCG strain but not in the *aotM* deletion strain (Figure 3B). The negative control *ppsAp* DNA could not be captured by the antibody in either WT BCG or *aotM* deletion mutant (Figure 3B). Taken together, AotM specifically binds to its promoter DNA region in *M. bovis* BCG in vivo and in vitro.

### 3.3. AotM Recognizes a 14-bp Palindrome Sequence Motif

To further identify the DNA-binding site of AotM, we performed a DNase I footprinting assay. The 1 μM purified AotM protein was co-incubated with the FAM-labeled fluorescent DNA probe *aotMp*, and then digested with DNase I. The DNA sequence located in the region −46 to −1 on the coding strand was clearly protected (Figure 3C). Interestingly, this region contains a 14-bp palindromic sequence (TGTCAATGTTGACA) formed by two inverted repeats (IR, 5′-TGTCAA-3′) (Figure 3D). Further EMSA assays confirmed the significance of this motif for specific recognition by AotM (Figure 3E, F). A series of DNA substrates in which one of the inverted repeats or both inverted repeats were mutated (Figure 3E). AotM was incapable of binding to the DNA substrates mutated within either of the inverted repeats (Figure 3F). A competition assay further confirmed the specificity of AotM binding with the core motif region. The cold (unlabeled) DNA substrate (P1) with the complete core motifs (Figure 3E) could competitively inhibit the binding of AotM to the FAM-labeled DNA substrate (P1) (Figure 3E and Figure S2). While, the DNA substrate (P2) mutated within the core motifs was failed to inhibit the binding of AotM to the FAM-labeled DNA substrate P1. Taken together, these results demonstrated that AotM recognizes a specific palindrome sequence motif.

### 3.4. AotM Affects the Expression of Genes Involved in Antioxidation in Mycobacterium bovis

**Figure 4:**
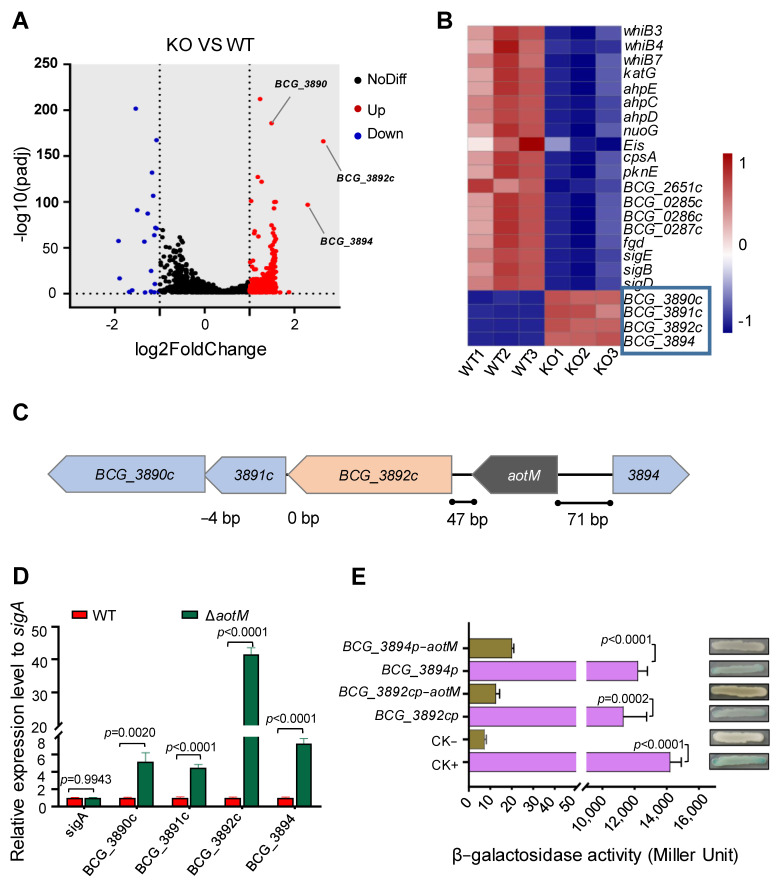
AotM regulates the expression of the adjacent genes. (**A**) The transcriptome difference between the wild-type *M. bovis* BCG and the *aotM* deletion mutant determined by RNA-seq was shown in the volcanic diagram. The significantly up-regulated- and the significantly downregulated genes were shown by red and blue spots, respectively. Genes that were not significantly changed were shown by black spots. (**B**) Heat map of the AotM-regulated differential expression profile for the antioxidant genes and adjacent genes. WT1, WT2, and WT3 represent separately three biological replicates of the genes in the wild-type strain. KO1, KO2, and KO3 represent separately three biological replicates of differentially expressed genes in the *aotM* knockout strain. Adjacent genes of *aotM* are indicated in the blue boxes. (**C**) Localization of the *aotM* on the genome in *M. bovis* BCG. The number at the bottom represents the number of separated bases between adjacent genes. (**D**) RT-qPCR was performed to detect the expression of the target gene in the Δ*aotM* deletion mutant and wild-type control strains. The relative gene expression levels were normalized using the *sigA* gene as an internal reference. The error bars represent the standard deviation of three biological replicates. A two-tailed unpaired Student’s *t*-test was used for statistical analysis using GraphPad Prism 8. *P*-values are indicated. (**E**) β-galactosidase activity assay. Schematic diagram of plasmids containing promoter-*lacZ* and promoter-*aotM*-*lacZ*, no promoter as negative control (CK−) and *hsp60* promoter as a positive control (CK+). All the plasmids were transformed into the *Escherichia coli*. The activity of β-galactosidase is expressed as Miller units. Data are expressed as the mean ± standard deviations of three independent replicates.

To identify the AotM-affected genes, we analyzed the differences in transcriptome between the Δ*aotM* deletion mutant and the WT strain. 377 genes were identified with significantly altered expression levels via filtering for differential genes with fold change (FC) values greater than 2. Of these, 352 genes were up-regulated, and 25 genes were down-regulated more than twofold (Figure 4A), suggesting a broad regulatory role of AotM in Mycobacterium bovis. Importantly, 19 genes involved in antioxidation, including *katG*, *pknE*, *sigE*, *BCG_0286c,* and *BCG_0287c* were significantly down-regulated in the Δ*aotM* deletion strains (Figure 4B and Table S4), suggesting that AotM might promote the expression of genes involved in antioxidation in *Mycobacterium bovis*.

In addition, the genes between *BCG_3890c* and *BGC_3894* loci (Figure 4C) were significantly up-regulated in the Δ*aotM* deletion strain (Figure 4B), which is consistent with the results in the RT-qPCR experiment (Figure 4D). Notably, *BGC_3892c* encoding a FAD-dependent oxidoreductase was up-regulated by approximately 40-fold in the Δ*aotM* deletion strain (Figure 4D), suggesting that AotM inhibits the expression of BCG_3892c.

### 3.5. AotM Negatively Regulates the Expression of the Adjacent Genes

**Figure 5:**
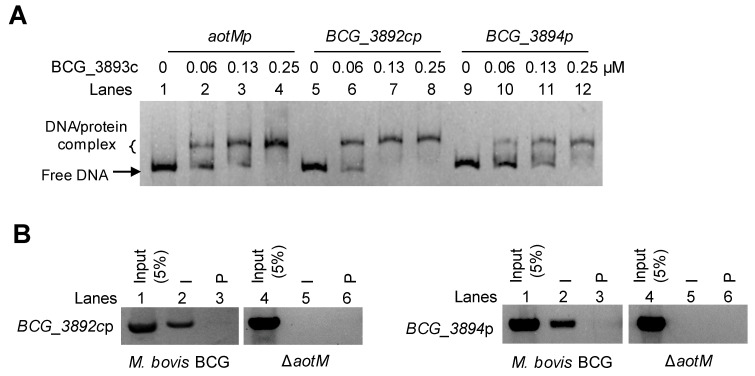
AotM binds to the promoters of the target genes. (**A**) EMSA of AotM binding to the promoters of the target genes. The mixtures were electrophoresed in a 5% native polyacrylamide gel. The 100 ng *aotM* promoter (*aotMp*, 500 bp, lanes 1–4), 100 ng *BCG_3892c* promoter (*BCG_3892cp*, 500 bp, lanes 5–8) and 100 ng *BCG_3894* promoter (*BCG_3894p*, 500 bp, lanes 9–12) were incubated with different concentrations (0–0.25 µM) of AotM protein. (**B**) ChIP assays for intracellular binding of AotM to the promoters of the target genes. The experiment was performed in wild-type and *aotM* knockout strains with post-immune serum (I) and pre-immune serum (P) to capture the complexes of protein and the promoters of the target genes.

Genomic location analysis showed that *aotM* is spaced 47-bp and 71-bp to the downstream adjacent gene *bcg_3892c* and the upstream adjacent gene *bcg_3894* (Figure 4C), respectively. Then, we tested whether AotM could bind to the promoter DNA of the adjacent genes in vitro. In the EMSA experiments, the increased shifted bands were clearly observed when the increasing concentrations of AotM were incubated with the promoters DNA *aotMp*, *bcg_3892cp,* or *bcg_3894p* (Figure 5A), indicating that AotM protein binds to the promoters of the adjacent target genes. Furthermore, we examined the in vivo DNA-binding ability of AotM to the promoters of adjacent genes by ChIP assay. In the wild-type BCG strain, the AotM antibody could capture both the complex of AotM with *bcg_3892cp* (Figure 5B, left panel) and the complex of AotM with *bcg_3894p* (Figure 5B, right panel). While the AotM antibody could not capture *bcg_3892cp* and *bcg_3894p* in the knockout strain, indicating that AotM indeed binds to *bcg_3892cp* and *bcg_3894p in vivo*. Taken together, these results suggest that transcription regulator AotM binds to the upstream regions of the adjacent target genes *bcg_3892c* and *bcg_3894* and exerts its regulatory functions.

Next, we wanted to know whether AotM acts as a repressor of the adjacent target genes. We performed the β-galactosidase assays using *lacZ* reporter plasmids. The *lacZ* gene was placed under the control of the promoter regions from the potential target genes, including *bcg_3892c* and *bcg_3894.* In the absence of *aotM*, both reporter strains showed high β-galactosidase activities. The β-galactosidase activities were significantly inhibited when *aotM* was expressed (Figure 4E), suggesting AotM negatively regulates the expression of the adjacent target genes. Taken together, AotM acts as a repressor to the adjacent genes.

### 3.6. Overexpression of BCG_3892c Leads to Increased Intracellular ROS Level and Reduced Growth of M. bovis

**Figure 6:**
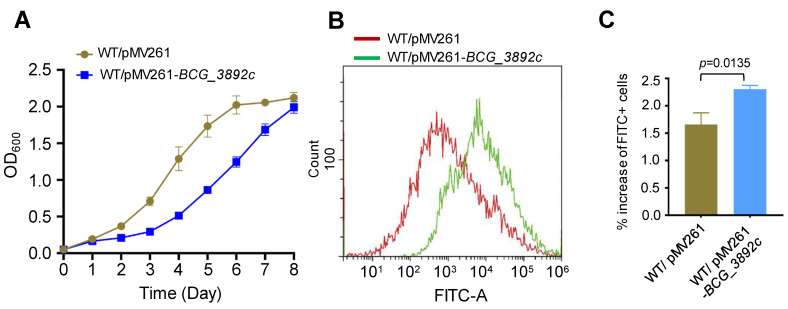
Overexpression of BCG_3892c increases the intracellular ROS levels in *M. bovis* BCG. (**A**) Detection of Wild-type *M. bovis* BCG strain with pMV261 (WT/pMV261) and the *BCG_3892c* overexpressing strain (WT/pMV261-*BCG_3892c*) growth in the 7H9 media. The results show the mean of three biological replicates, and the error bars indicate the standard deviation of three biological replicates. (**B**) The intracellular ROS levels of the strains were analyzed by flow cytometry. The horizontal coordinate FITC-A represents the detected fluorescence value, and the vertical coordinate represents the detected number of bacteria in each sample. (**C**) The fluorescence signal levels detected in the strains were statistically analyzed. The error bars represent the standard deviation of the three biological replicates. A two-tailed unpaired Student’s *t*-test was used for statistical analysis using GraphPad Prism 8. *P*-values are indicated.

AotM strongly inhibits the expression of *bcg_3892c* gene (Figure 4D,E). Then, we were curious about the effects of the dysfunctional expression of *bcg_3892c*. To test this, we overexpressed *bcg_3892c* in the WT BCG strain using the replicative vector pMV261. An approximately 22-fold upregulation of the transcription level of *bcg_3892c* was confirmed in the overexpression strain by the RT-qPCR experiment (Figure S3). Interestingly, overexpression of *bcg_3892c* partially inhibited the growth of *M. bovis* (Figure 6A). The *bcg_3892c* gene encodes a putative FAD-dependent oxidoreductase. Oxidation processes could usually affect intracellular ROS levels [37,38]. To test whether the intracellular ROS levels were changed, we performed a dichloro-dihydro-fluorescein diacetate (DCFH-DA) assay, which is widely used in the detection of reactive oxygen species (ROS) [36]. The fluorescent probe DCFH-DA was incubated with the active bacterial cells, and then the intracellular ROS levels were indicated by the intensity of the fluorescent signal (FITC-A). Interestingly, we found that the fluorescence signal was increased by 41% in the *BCG_3892c* overexpression strain compared to the control strain with the same bacterial population (Figure 6B,C), suggesting that overexpression of *BCG_3892c* increases the intracellular ROS production. In conclusion, these results suggest that overexpression of BCG_3892c could reduce the growth of *M. bovis,* probably due to the increased intracellular ROS production.

## 4. Discussion

Antioxidation is particularly important for the survival of intracellular pathogens, including Mtb, which is often exposed to ROS in the host cells during infection. In this study, we identified *M. bovis* AotM as a novel redox sensor required for mycobacterial growth in the presence of hydrogen peroxide. The transcriptome data showed that AotM broadly affects the expression of genes in *M. bovis*. Loss of *aotM* gene leads to altered transcriptional profiles, with 352 genes significantly up-regulated and 25 genes significantly down-regulated. Interestingly, we found that 19 genes involved in antioxidation, including *katG*, *pknE*, *sigE*, *bcg_0286c*, and *bcg_0287c*, were significantly down-regulated in the Δ*aotM* deletion strains (Figure 4B). In bacteria, KatG proteins have peroxidase activity and are considered to be involved in the antioxidant process. In Mtb, the *katG* gene could be induced under oxidative stress. The Mtb KatG has peroxidase activity [39], which facilitates the degradation of peroxides produced by NADPH oxidases in macrophages during Mtb infection [14]. PknE, a serine/threonine protein kinase (STPK), plays a very important role in mycobacteria facing nitrogen and ROS stress [40]. PknE could respond to the oxidative stress of NO in the environment, which helps the bacteria clear intracellular free radicals. The loss of *pknE* leads to the increased resistance of Mtb to NO [41]. Also, the sigma factor E encoded by *sigE* could be induced by the oxidative stress during bacterial replication. SigE could regulate mycobacterial cells to maintain intracellular redox potential [42]. *BCG_0286c* and *BCG_0287c* encode succinate dehydrogenases, which promote the antioxidant growth of the bacterium in concert with the Fgd-F420 system [43,44]. In this study, we found that *M. bovis* BCG_3892c, a putative oxidoreductase, is involved in intracellular ROS production. Overexpression of *BCG_3892c* leads to increased intracellular ROS levels and reduced bacterial growth (Figure 6). However, the specific function and metabolic pathway of BCG_3892c are not clear, while AotM indeed acts as a repressor to *BCG_3892c.* In the absence of AotM, *BCG_3892c* was up-regulated by 40-fold. Our findings suggest that some of the metabolic enzymes associated with ROS production could be strictly regulated in mycobacteria. Interestingly, a recent study showed that *M. smegmatis* SdrR, a LysR-type regulator, was able to modulate the mycobacterial tolerance to oxidative stress via regulating the expression of a short-chain oxidoreductase SDR encoded by *MSMEG_5714* [45]. Unlike AotM, which plays an essential role in the mycobacterial resistance to hydrogen peroxide, SdrR exhibits a negative effect on the resistance of *M. smegmatis* to hydrogen peroxide. Loss of *sdrR* indeed leads to increased expression of the short-chain oxidoreductase SDR, which facilitates the survival of *M. smegmatis* in the presence of hydrogen peroxide [45]. These results imply that mycobacteria might use multiple regulatory pathways to control the oxidoreductases and promote bacterial growth under oxidative stress.

In bacteria, transcription regulators containing cysteine residues could be used to sense oxidative signals. For example, MosR senses H_2_O_2_ stress via its N-terminal two cysteines (Cys10 and Cys12) in Mtb, and the oxidized MosR regulates the expression of a short-chain dehydrogenase encoded by *rv1050* [11]. Similarly, OxyS in Mtb senses H_2_O_2_ via cysteines (Cys25) and subsequently regulates the expression of the catalase KatG [11,46]. Also, CmtR senses H_2_O_2_ stress via cysteine (Cys24), and it physically interacts with the zinc uptake regulator Zur to promote the expression of *esx-3* operon, thereby enhancing the accumulation of Zn^2+^ in *M. bovis* and triggering the detoxification of ROS [27]. AotM protein contains three cysteines at positions 142, 160, and 187. Then, we hypothesized that AotM might also sense oxidative stress via the formation of a disulfide bond. However, we could not observe any changes to the DNA-binding activity of AotM in the EMSA experiments using the cysteine-mutated AotM protein in the presence of hydrogen peroxide, suggesting that AotM sensing oxidative signals could be cysteine-independent. In addition, some oxidative stress transcription factors could sense ROS in other ways. For example, the iron-sulfur [2Fe-2S] cluster in SoxR of *P. aeruginosa* [26] and the histidines in PerR of *B. subtilis* [47,48] were used for sensing the oxidative signals. In some cases, methionine could regulate oxidative stress in nitrogen metabolism in *S. cerevisiae* [49]. Therefore, the key residues of AotM for sensing oxidative stress need to be further identified in the future.

## 5. Conclusions

**Figure 7:**
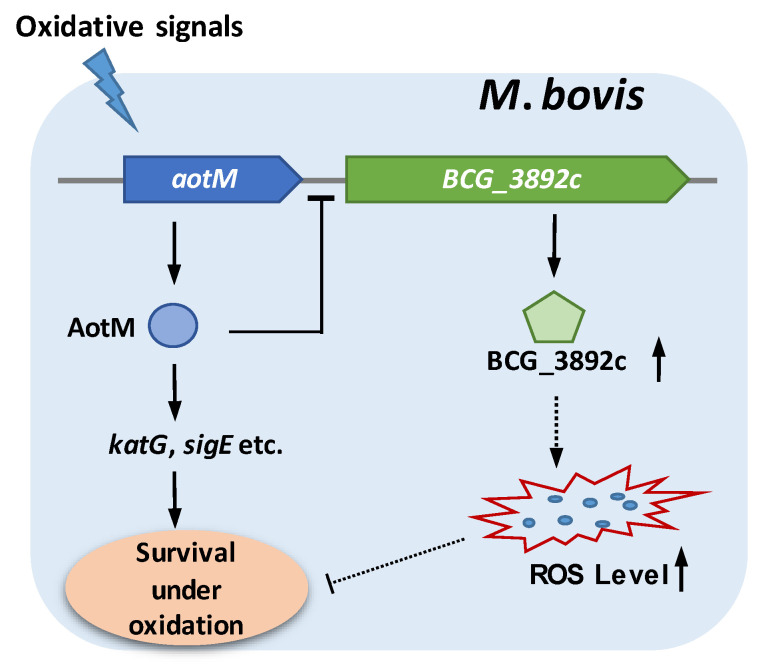
Model of AotM regulation. *aotM* gene could respond to the oxidative signals. The transcription regulator AotM promotes the expression of the antioxidant genes, including *katG, sigE*, etc., facilitating the survival of *M bovis* under oxidative stress. Meanwhile, AotM inhibits the expression of *BCG_3892c*, which encodes a dehydrogenase. Overexpression of BCG_3892c leads to the increased production of ROS, which inhibits mycobacterial growth. The dashed arrow or line represent the intricate correlation which remains to be further investigated.

ROS plays a very important role in resisting the invasion of pathogens in the organism. When the pathogens invade the hosts and form foci, the local level of ROS in the foci will increase dramatically to cause oxidative damage to the pathogens as a way of resisting the further invasion of the pathogens [50]. *Mycobacterium tuberculosis* complex species (MTBC), including *M. bovis*, as successful intracellular pathogens, have evolved unique antioxidant growth strategies with many oxidative stress-responsive transcription regulators, which usually play important roles in coping with the oxidative environment of host cells. In this study, we characterized a novel oxidative stress-responsive transcription regulator AotM, which is required for the survival of *M. bovis* in the presence of hydrogen peroxide. AotM specifically recognizes a 14-bp palindrome sequence motif and negatively regulates the expression of the target gene *BCG_3892c* encoding a FAD-dependent oxidoreductase (Figure 7). Overexpression of the oxidoreductase leads to increased intracellular ROS levels and reduced bacterial growth. Therefore, AotM might restrict intracellular ROS production by repressing the expression of the oxidoreductase, facilitating *M. bovis* to survive under oxidative stress (Figure 7). Our findings indicate the existence of a novel antioxidant regulation pathway and provide new insights into mycobacterial adaptation to oxidative stress.

## Data Availability

The original contributions presented in the study are included in the article/Supplementary Materials, further inquiries can be directed to the corresponding authors.

## Supplementary Materials

The following supporting information can be downloaded at: https://www.mdpi.com/article/10.3390/biomedicines12081872/s1, Figure S1: *aotM* expression in recombinant strains; Figure S2: AotM specifically binds to the core motif region; Figure S3: *BCG_3892c* expression in recombinant strains; Table S1: Bacterial strains used in this work; Table S2: Plasmids used in this work; Table S3: Oligonucleotides used in this work; Table S4: Functional presentation of antioxidant genes affected by AotM deletion. References [51,52,53,54,55,56,57,58,59,60,61,62,63,64,65,66,67] are cited in the supplementary materials.
