# Supplementary material for: Characterization of a Novel Oxidative Stress Responsive Transcription Regulator in Mycobacterium bovis
Source: Biomedicines. 2024 Aug 16;12(8):1872. doi: 10.3390/biomedicines12081872 (PMC11351531; doi:10.3390/biomedicines12081872)
Supplement: Supplementary file 1 [file biomedicines-12-01872-s001.zip › biomedicines-3131800-supplementary.pdf]

Supplementary Information

Characterization of a Novel Oxidative Stress Responsive Transcription Regulator in *Mycobacterium bovis*

Qiang Jiang [1], Rong Hu [1], Feng Liu [1], Feng Huang [1], Lei Zhang [2,*] and Hua Zhang [1,*]

1 College of Life Science and Technology, Huazhong Agricultural University, Wuhan 430070, China
2 College of Veterinary Medicine, Huazhong Agricultural University, Wuhan 430070, China
* Correspondence: zhanglei2023@mail.hzau.edu.cn (L.Z.); zhanghua@mail.hzau.edu.cn (H.Z.)

Supplementary Figures

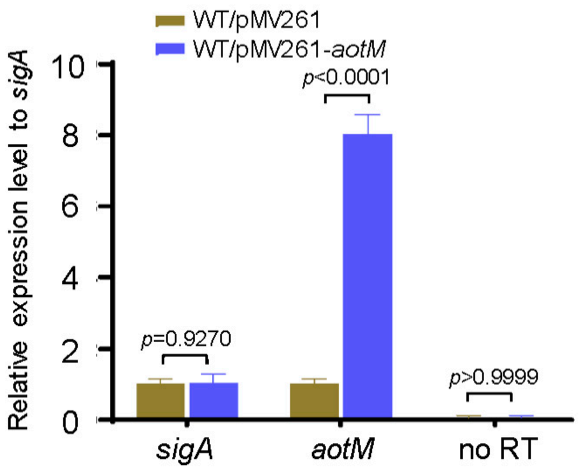

Figure S1. *aotM* expression in recombinant strains.

The quantitative real time-PCR assays were performed for testing the transcription level of *aotM* in the Wild-type *M. bovis* BCG strain (WT/pMV261) and *aotM* overexpression *M. bovis* BCG strain (WT/pMV261-*aotM*). *sigA* was used as the reference. Error bars represent the standard deviation of the three biological replicates. Two-tailed unpaired Student's *t*-test was used for statistical analysis using GraphPad Prism 8. *P*-values are indicated.

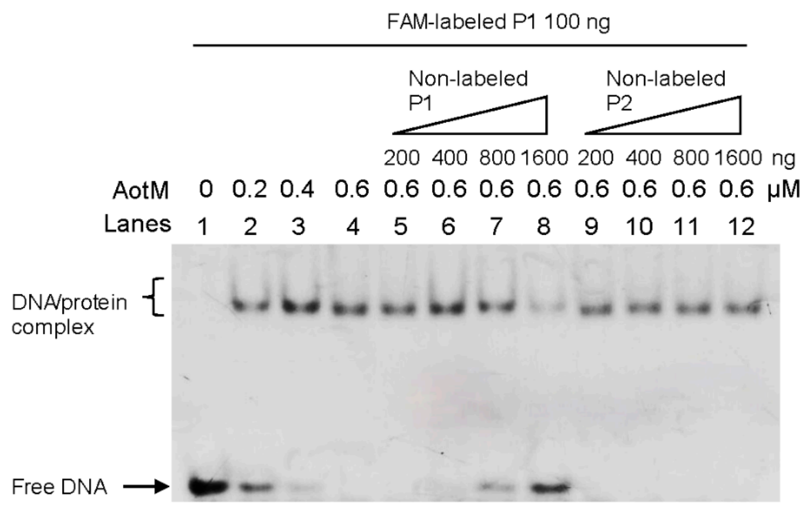

Figure S2. AotM specifically binds to the core motif region.

Competitive assays for the specific DNA binding activity of AotM with the short core motif region. Experiments were carried out to test the specificity of DNA binding. 100 ng FAM-labeled DNA substrate P1 was co-incubated with 0.2-0.6 μM of AotM protein (lanes 2-4). Different amounts of unlabeled P1 DNA substrate (lanes 5-8) or IR mutated DNA substrate P2 (lanes 9-12) at 2-, 4-, 8-, and 16-fold excess compared with the FAM-labeled P1 DNA substrate were added into the reaction mixture.

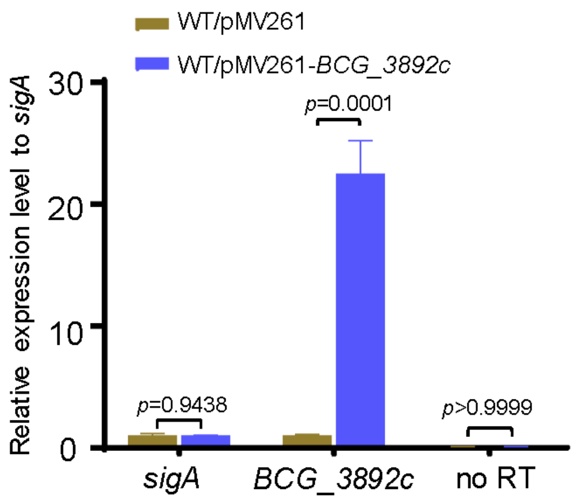

Figure S3. *BCG_3892c* expression in recombinant strains.

The quantitative real time-PCR assays were performed for testing the transcription level of *BCG_3892c* in the WT *M. bovis* BCG strain (WT/pMV261) and the *BCG_3892c* overexpression strain (WT/pMV261-*BCG_3892c*). *sigA* was used as the reference. Error bars represent the standard deviation of the three biological replicates. Two-tailed unpaired Student's *t*-test was used for statistical analysis using GraphPad Prism 8. *P*-values are indicated.

Supplementary Tables

**Strain**	**Relevant genotape or feature**	**Source**
E. coli		
E.coli DH5α	Host for plasmid construction	TaKaRa
E.coli BL21	Host for protein expression	TaKaRa
***M.bovis* BCG**		
WT BCG	Wide-type *M.bovis* BCG	This study
BCG/pMV261	BCG with pMV261, Kan^R	This study
BCG/*aotM::hyg*	BCG with *aotM* replaced by *hyg*, hyg^R	This study
BCG/pMindD-*aotM*	Complementary strains after aotM deletion	This study
BCG/pMV261-*aotM*	*aotM*-overexpression Strain, Kan^R	This study
BCG/pMV261-*BCG_3892c*	*BCG_3892c*-overexpression Strain, Kan^R	This study

Table S1. Bacterial strains used in this work.
The annotations hyg^R and kan^R indicate that the strain is resistant to the antibiotics hygromycin and kanamycin, respectively.

Plasmids	Characteristics	Source
**pET28a**	*Kan*[R], containing the lacZ operon, T7 promoter, and His-Tag	Novagen
pET28a-*aotM*	*aotM* inserted in *Eco*RI-*Xba*I of pET28a	This study
**pMindD**	*Kan*[R], containing ColiE1 and pAL5000 replicon	This study
pMindD-*aotM*	*aotM* inserted in *Eco*RI-*Xba*I of pMindD	This study
**pMV261**	*Kan*[R], containing pAL5000 replicon	This study
pMV261-*aotM*	*aotM* inserted in *Eco*RI-*Xba*I of pMV261	This study
pMV261-*BCG_3892c*	*BCG_3892c* inserted in *Eco*RI-*Xba*I of pMV261	This study
**pMV261-*lacZ***	*Kan*[R], containing the *lacZ* operon	This study
pMV261-*BCG_3892cp-lacZ*	*BCG_3892cp* inserted in *Eco*RI-*Xba*I of pMV261-*lacZ*	This study
pMV261-*BCG_3892cp-aotM-lacZ*	*BCG_3892cp-aotM* inserted in *Eco*RI-*Xba*I of pMV261-*lacZ*	This study
pMV261-*BCG_3894p-lacZ*	*BCG_3894p* inserted in *Eco*RI-*Xba*I of pMV261-*lacZ*	This study
pMV261-*BCG_3894p-aotM-lacZ*	*BCG_3894p-aotM* inserted in *Eco*RI-*Xba*I of pMV261-*lacZ*	This study

Table S2. Plasmids used in this work**.**
The *kan*[R] indicates that the strain is resistant to the antibiotics kanamycin.

**Name**	**Sequence (5’ to 3’)**	**Use**
aotM-F	ATTCGAATTCAAATGGTCCGGCCCCCGCAGAC	Clone, expression
aotM-R	CCGATCTAGATTATGACGAAACTGTGAGGG	Clone, expression
*aotM*up-F	CGCGTTAATTAACCCGGTTATGGGGCGACACT	Gene deletion, PCR validation
*aotM*up-R	GCGCGCACTAGTCACGACTGCAAGCTATCGTCAAT	Gene deletion, PCR validation
*aotM*dn-F	CGCGAAGCTTTTGACAAAACATCGAGCGCG	Gene deletion, PCR validation
*aotM*dn-R	ATAGGCTAGCATGCGCAAATAGCGATCGCG	Gene deletion, PCR validation
BCG_3892c-F	GCATGAATTCGGATGACTGGTTATGACGCGAT	Clone, expression
BCG_3892c-R	ATCGTCTAGACTACCGACCACTCAAAACGC	Clone, expression
aotMp-F	ATGAAAACAGCGGCAGCAGGAT	EMSA, ChIP
aotMp-R	AATTAGGTAGCCTCGACGCCCTGG	EMSA, ChIP
BCG_3892cp-F	GCGCTCTCGTTTCCTTATGACGTT	EMSA
BCG_3892cp-R	TATATCGTCCAGACACGCGGTCCGCA	EMSA
BCG_3894p-F	ATATCGCCGCAACGACGCAATCTT	EMSA
BCG_3894p-R	ATATATACGATCCGCCGGCCGGCCTG	EMSA
ppsAp-F	ATCGGCGTCATTTCCGATGGTGAG	EMSA, ChIP
ppsAp-R	ATATAGATCGGGGTCCACCTCGTCA	EMSA, ChIP
P1-F (FAM-labeled)	CCAACAGTTTTGTCAATGTTGACAGAACGAAAAT	EMSA
P1-R	ATTTTCGTTCTGTCAACATTGACAAAACTGTTGG	EMSA
P2-F	CCAACAGTTTGAGTCCTGCCACTCGAACGAAAAT	EMSA
P2-R	ATTTTCGTTCGAGTGGCAGGACTCAAACTGTTGG	EMSA
P3-F	CCAACAGTTTGAGTCCTGTTGACAGAACGAAAAT	EMSA
P3-R	ATTTTCGTTCTGTCAACAGGACTCAAACTGTTGG	EMSA
P4-F	CCAACAGTTTTGTCAATGCCACTCGAACGAAAAT	EMSA
P4-R	ATTTTCGTTCGAGTGGCATTGACAAAACTGTTGG	EMSA
RT-*aotM*-F	GCGGCTATCTTCTCGTTTCC	RT-qPCR

RT-*aotM*-R	GATATCGCAGTTACGTCGCC	RT-qPCR
RT-*BCG_3890c*-F	GCGTTACTGGTATCGGGACT	RT-qPCR
RT-*BCG_3890c*-R	AGTTCCAGTACGCATCGACA	RT-qPCR
RT-*BCG_3891c*-F	GTTGGGCGTTGAATGGACAT	RT-qPCR
RT-*BCG_3891c*-R	CGTCATCAACCTCAGCCAAG	RT-qPCR
RT-*BCG_3892c*-F	GATCTGGAGGTGATGTCGGT	RT-qPCR
RT-*BCG_3892c*-R	CGCTGACCGTTCGAATTCAT	RT-qPCR
RT-*BCG_3894*-F	GCTGGGGTGGTACTTCAACA	RT-qPCR
RT-*BCG_3894*-R	GCTCACGAAATACAGCCAGG	RT-qPCR
RT-*cmtR*-F	TGCTGGATGGCGTTTGCT	RT-qPCR
RT-*cmtR*-R	GGCCCTCATAGGTTGCGACTA	RT-qPCR
RT-*fbpB*-F	CGCGACATCAAGGTTCAGTT	RT-qPCR
RT-*fbpB*-R	CCGGCATGACTATCGACAGT	RT-qPCR
RT-*sigA*-F	TCGCGCCTACCTCAAACAG	RT-qPCR
RT-*sigA*-R	CGTACAGGCCAGCCTCGAT	RT-qPCR

Table S3. Oligonucleotides used in this work.
Restriction sites are underlined.

ORF	gene	Function	References
BCG_3486	*whiB3*	redox-sensing transcriptional regulator	51
BCG_3740c	*whiB4*	redox-sensing transcriptional regulator	52
BCG_3221c	*whiB7*	redox-sensing transcriptional regulator	53
BCG_1947c	*katG*	catalase-peroxidase	54
BCG_2447	*ahpC*	Offer defense against oxidative stress via NADH-dependent peroxidase and peroxynitrite reductase	55,56, 57
BCG_2448	*ahpD*		
BCG_2256c	*ahpE*	Peroxide detoxification	58, 59
BCG_3174	*nuoG*	NADH dehydrogenase I	60
BCG_2432c	*eis*	enhanced intracellular survival protein	61
BCG_3548	cpsA	Antagonizes host innate immunity by inhibiting NADPH oxidase and LAP	62
BCG_1782	*pknE*	involved in NO-related oxidative stress	63
BCG_2651c	*BCG_2651c*	universal stress protein	64
BCG_0285c	*BCG_0285c*	Modulates oxidative phosphorylation and central metabolism by maintaining membrane potential for energy production	65
BCG_0286c	*BCG_0286c*		
BCG_0287c	*BCG_0287c*		
BCG_0446	*fgd*	maintaining redox homeostasis and latency reactivation	66, 67
BCG_2723	*sigB*	tress-induced sigma factor, transcriptionally regulates the expression of different anti-oxidants	68, 69, 70
BCG_3484c	*sigD*		
BCG_1281	*sigE*		

Table S4. Functional presentation of antioxidant genes affected by AotM deletion.

References of Table S4

51. Saini, V.; Farhana, A.; Steyn, A.J. *Mycobacterium tuberculosis* WhiB3: a novel iron-sulfur cluster protein that regulates redox homeostasis and virulence. *Antioxid Redox Signal*. **2012**, 16, 687-697.
52. Wu, J.; Ru, H.W.; Xiang, Z.H.; et al. WhiB4 Regulates the PE/PPE Gene Family and is Essential for Virulence of *Mycobacterium marinum*. *Sci Rep*. **2017**, 7, 3007.
53. Burian, J.; Ramón-García, S.; Sweet, G.; Gómez-Velasco, A.; Av-Gay, Y.; Thompson, C.J. The mycobacterial transcriptional regulator *whiB7* gene links redox homeostasis and intrinsic antibiotic resistance. *J Biol Chem*. **2012**, 287, 299-310.
54. Machado, P.; Bizarro, C.V.; Basso, L.A. Resistance Reversed in KatG Mutants of *Mycobacterium tuberculosis*. *Trends Microbiol*. **2019**, 27, 655-656.

55. Hillas, P.J.; Del, Alba, F.S.; Oyarzabal, J.; Wilks, A.; Ortiz, De, Montellano, P.R. The AhpC and AhpD antioxidant defense system of *Mycobacterium tuberculosis*. *J BiolChem*, **2000**, 275, 18801-18809.
56. Springer, B.; Master, S.; Sander, P.; et al. Silencing of oxidative stress response in *Mycobacterium tuberculosis*: expression patterns of *ahpC* in virulent and avirulent strains and effect of *ahpC* inactivation. *Infect Immun*. **2001**, 69, 5967-5973.
57. Lee, H.N.; Lee, N.O.; Han, S.J.; Ko, I.J.; Oh, J.I. Regulation of the *ahpC* gene encoding alkyl hydroperoxide reductase in M*ycobacterium smegmatis*. *PLoS One*. **2014**, 9, e111680.
58. Jaeger, T. Peroxiredoxin systems in mycobacteria. *Subcell Biochem.* **2007**, 44, 207-217.
59. Perkins, A.; Nelson, K.J.; Parsonage, D.; Poole, L.B.; Karplus, P.A. Peroxiredoxins: guardians against oxidative stress and modulators of peroxide signaling. *Trends Biochem Sc*i. **2015**, 40, 435-445.
60. Gengenbacher, M.; Nieuwenhuizen, N.; Vogelzang, A.; et al. Deletion of *nuoG* from the Vaccine Candidate *Mycobacterium bovis* BCG Δ*ureC*::*hly* Improves Protection against Tuberculosis. *mBio*. **2016**, 7, 00679-16.
61. Punetha, A.; Ngo, H.X.; Holbrook, S.Y.L.; et al. Structure-Guided Optimization of Inhibitors of Acetyltransferase Eis from *Mycobacterium tuberculosis. ACS Chem Biol*. **2020**, 15, 1581-1594.
62. Köster, S.; Upadhyay, S.; Chandra, P.; et al. *Mycobacterium tuberculosis* is protected from NADPH oxidase and LC3-associated phagocytosis by the LCP protein CpsA. *Proc Natl Acad Sci USA*. **2017**, 114, E8711-E8720.
63. Jayakumar, D.; Jacobs, W.R J.r.; Narayanan, S. Protein kinase E of *Mycobacterium tuberculosis* has a role in the nitric oxide stress response and apoptosis in a human macrophage model of infection. *Cell Microbiol*. **2008**, 10, 365-374.
64. Jia, Q.; Hu, X.; Shi, D.; et al. Universal stress protein Rv2624c alters abundance of arginine and enhances intracellular survival by ATP binding in mycobacteria. *Sci Rep*. **2016**, 6, 35462.
65. Knapp, G.S.; Lyubetskaya, A.; Peterson, M.W.; et al. Role of intragenic binding of cAMP responsive protein (CRP) in regulation of the succinate dehydrogenase genes Rv0249c-Rv0247c in TB complex mycobacteria. *Nucleic Acids Res*. **2015**, 43, 5377-5393.
66. Gurumurthy, M.; Rao, M.; Mukherjee, T.; et al. A novel F (420) -dependent anti-oxidant mechanism protects *Mycobacterium tuberculosis* against oxidative stress and bactericidal agents. *Mol Microbiol*. **2013**, 87, 744-755.
67. Nguyen, Q.T.; Trinco, G.; Binda, C.; Mattevi, A.; Fraaije, M.W. Discovery and characterization of an F420-dependent glucose-6-phosphate dehydrogenase (Rh-FGD1) from Rhodococcus jostii RHA1. *Appl Microbiol Biotechnol*. **2017**, 101, 2831-2842.
68. Rodrigue, S.; Provvedi, R.; Jacques, P.E.; Gaudreau, L.; Manganelli, R. The sigma factors of *Mycobacterium tuberculosis*. *FEMS Microbiol Rev*. **2006**, 30, 926-941.
69. Raman, S.; Hazra, R.; Dascher, C.C.; Husson, R.N. Transcription regulation by the *Mycobacterium tuberculosis* alternative sigma factor SigD and its role in virulence. *J Bacteriol*. **2004**, 186, 6605-6616.
70. Manganelli, R.; Cioetto-Mazzabò, L.; Segafreddo, G.; et al. SigE: A master regulator of *Mycobacterium tuberculosis*. *Front Microbiol*. **2023**, 14, 1075143.
